# Supplementary material for: Characterization of the Soil Bacterial Community from Selected Boxwood Gardens across the United States
Source: Microorganisms. 2022 Jul 26;10(8):1514. doi: 10.3390/microorganisms10081514 (PMC9330173; doi:10.3390/microorganisms10081514)
Supplement: Supplementary file 1 [file microorganisms-10-01514-s001.zip › microorganisms-1770539-supplementary.pdf]

## Supplemental Tables and Figures

Table S1. Sample and sequencing metadata

| Flowcell number | Run number | Samples | Barcodes | Year | Month | State | Site | Reads (qcat demultiplexed) | Reads (filtered) | Read filtering rate % |
|---|---|---|---|---|---|---|---|---|---|---|
| FAK92027 | 2 | 1705_CA1 | barcode01 | 2017 | 5 | California | CA1 | 484,460 | 151,999 | 31.37 |
| | | 1705_CA2 | barcode02 | 2017 | 5 | California | CA2 | 563,698 | 135,539 | 24.04 |
| | | 1705_CA3 | barcode03 | 2017 | 5 | California | CA3 | 433,826 | 142,795 | 32.92 |
| | | 1705_IL1 | barcode04 | 2017 | 5 | Illinois | IL1 | 406,275 | 109,979 | 27.07 |
| | | 1705_IL2 | barcode05 | 2017 | 5 | Illinois | IL2 | 390,758 | 118,241 | 30.26 |
| | | 1705_IL3 | barcode06 | 2017 | 5 | Illinois | IL3 | 555,953 | 184,678 | 33.22 |
| | | 1705_NYNE | barcode07 | 2017 | 5 | New York | NYNE | 468,841 | 129,559 | 27.63 |
| | | 1705_NYNW | barcode08 | 2017 | 5 | New York | NYNW | 377,868 | 114,876 | 30.4 |
| | | 1705_NYSE | barcode09 | 2017 | 5 | New York | NYSE | 292,747 | 94,962 | 32.44 |
| | | 1705_SC1 | barcode10 | 2017 | 5 | South Carolina | SC1 | 313,001 | 121,717 | 38.89 |
| | | 1705_SC2 | barcode11 | 2017 | 5 | South Carolina | SC2 | 411,193 | 140,136 | 34.08 |
| | | 1705_SC3 | barcode12 | 2017 | 5 | South Carolina | SC3 | 256,112 | 116,416 | 45.46 |
| | 3 | 1705_VAC1 | barcode01 | 2017 | 5 | Virginia | C1 | 210,870 | 61,510 | 29.17 |
| | | 1705_VAC2 | barcode02 | 2017 | 5 | Virginia | C2 | 1,570 | 435 | 27.71 |
| | | 1705_VAC3 | barcode03 | 2017 | 5 | Virginia | C3 | 162,283 | 46,950 | 28.93 |
| | | 1705_VAM1 | barcode04 | 2017 | 5 | Virginia | M1 | 271,738 | 113,837 | 41.89 |
| | | 1705_VAM2 | barcode05 | 2017 | 5 | Virginia | M2 | 287,569 | 89,192 | 31.02 |
| | | 1705_VAM3 | barcode06 | 2017 | 5 | Virginia | M3 | 366,969 | 125,807 | 34.28 |
| | | 1711_CA1 | barcode07 | 2017 | 11 | California | CA1 | 279,127 | 71,938 | 25.77 |
| | | 1711_CA2 | barcode08 | 2017 | 11 | California | CA2 | 198,841 | 65,385 | 32.88 |
| | | 1711_CA3 | barcode09 | 2017 | 11 | California | CA3 | 179,352 | 46,051 | 25.68 |
| | | 1711_IL1 | barcode10 | 2017 | 11 | Illinois | IL1 | 163,679 | 48,242 | 29.47 |
| | | 1711_IL2 | barcode11 | 2017 | 11 | Illinois | IL2 | 266,657 | 70,213 | 26.33 |
| | | 1711_IL3 | barcode12 | 2017 | 11 | Illinois | IL3 | 93,894 | 25,343 | 26.99 |
| FAK96961 | 1 | 1711_NYNE | barcode01 | 2017 | 11 | New York | NYNE | 514,521 | 178,249 | 34.64 |

| | | | | | | | | | |
|---|---|---|---|---|---|---|---|---|---|
| | 1711_NYNW | barcode02 | 2017 | 11 | New York | NYNW | 735,837 | 50,120 | 6.81 |
| | 1711_NYSE | barcode03 | 2017 | 11 | New York | NYSE | 320,039 | 112,684 | 35.21 |
| | 1711_NY1 | barcode04 | 2017 | 11 | New York | NY1 | 460,301 | 15,710 | 3.41 |
| | 1711_NY2 | barcode05 | 2017 | 11 | New York | NY2 | 584,159 | 190,958 | 32.69 |
| | 1711_NY3 | barcode06 | 2017 | 11 | New York | NY3 | 487,547 | 192,230 | 39.43 |
| | 1711_SC1 | barcode07 | 2017 | 11 | South Carolina | SC1 | 409,716 | 139,265 | 33.99 |
| | 1711_SC2 | barcode08 | 2017 | 11 | South Carolina | SC2 | 542,919 | 215,707 | 39.73 |
| | 1711_SC3 | barcode09 | 2017 | 11 | South Carolina | SC3 | 339,762 | 144,057 | 42.4 |
| | 1711_VAC1 | barcode10 | 2017 | 11 | Virginia | C1 | 374,443 | 174,402 | 46.58 |
| | 1711_VAC2 | barcode11 | 2017 | 11 | Virginia | C2 | 318,744 | 126,507 | 39.69 |
| | 1711_VAC3 | barcode12 | 2017 | 11 | Virginia | C3 | 252,732 | 109,320 | 43.26 |
| 2 | 1711_VAM1 | barcode01 | 2017 | 11 | Virginia | M1 | 388,079 | 136,970 | 35.29 |
| | 1711_VAM2 | barcode02 | 2017 | 11 | Virginia | M2 | 302,728 | 109,643 | 36.22 |
| | 1711_VAM3 | barcode03 | 2017 | 11 | Virginia | M3 | 233,240 | 86,730 | 37.18 |
| | 1806_CA1 | barcode04 | 2018 | 6 | California | CA1 | 318,093 | 151,798 | 47.72 |
| | 1806_CA2 | barcode05 | 2018 | 6 | California | CA2 | 384,553 | 147,795 | 38.43 |
| | 1806_CA3 | barcode06 | 2018 | 6 | California | CA3 | 559,779 | 199,960 | 35.72 |
| | 1806_IL1 | barcode07 | 2018 | 6 | Illinois | IL1 | 365,124 | 125,683 | 34.42 |
| | 1806_IL2 | barcode08 | 2018 | 6 | Illinois | IL2 | 354,173 | 135,480 | 38.25 |
| | 1806_IL3 | barcode09 | 2018 | 6 | Illinois | IL3 | 278,987 | 112,239 | 40.23 |
| | 1806_NYNE | barcode10 | 2018 | 6 | New York | NYNE | 285,463 | 92,782 | 32.5 |
| | 1806_NYNW | barcode11 | 2018 | 6 | New York | NYNW | 440,410 | 116,212 | 26.39 |
| | 1806_NYSE | barcode12 | 2018 | 6 | New York | NYSE | 208,272 | 70,774 | 33.98 |
| 3 | 1806_NY1 | barcode01 | 2018 | 6 | New York | NY1 | 230,439 | 70,455 | 30.57 |
| | 1806_NY2 | barcode02 | 2018 | 6 | New York | NY2 | 299,596 | 68,726 | 22.94 |
| | 1806_NY3 | barcode03 | 2018 | 6 | New York | NY3 | 60,219 | 4,689 | 7.79 |
| | 1806_SC1 | barcode04 | 2018 | 6 | South Carolina | SC1 | 288,529 | 93,567 | 32.43 |
| | 1806_SC2 | barcode05 | 2018 | 6 | South Carolina | SC2 | 259,089 | 76,568 | 29.55 |
| | 1806_SC3 | barcode06 | 2018 | 6 | South Carolina | SC3 | 330,778 | 124,083 | 37.51 |
| | 1806_VAC1 | barcode07 | 2018 | 6 | Virginia | C1 | 323,776 | 92,037 | 28.43 |
| | 1806_VAC2 | barcode08 | 2018 | 6 | Virginia | C2 | 262,287 | 85,092 | 32.44 |

| | | | | | | | | | | |
|---|---|---|---|---|---|---|---|---|---|---|
| | | 1806_VAC3 | barcode09 | 2018 | 6 | Virginia | C3 | 223,785 | 67,595 | 30.21 |
| | | 1806_VAM1 | barcode10 | 2018 | 6 | Virginia | M1 | 235,557 | 74,118 | 31.46 |
| | | 1806_VAM2 | barcode11 | 2018 | 6 | Virginia | M2 | 375,500 | 113,940 | 30.34 |
| | | 1806_VAM3 | barcode12 | 2018 | 6 | Virginia | M3 | 318,890 | 93,695 | 29.38 |
| FAK96899 | 1 | 1811_CA1 | barcode01 | 2018 | 11 | California | CA1 | 413,509 | 135,074 | 32.67 |
| | | 1811_CA2 | barcode02 | 2018 | 11 | California | CA2 | 545,771 | 183,650 | 33.65 |
| | | 1811_CA3 | barcode03 | 2018 | 11 | California | CA3 | 252,149 | 94,622 | 37.53 |
| | | 1811_VAC1 | barcode04 | 2018 | 11 | Virginia | C1 | 654,665 | 162,116 | 24.76 |
| | | 1811_VAC2 | barcode05 | 2018 | 11 | Virginia | C2 | 495,873 | 138,932 | 28.02 |
| | | 1811_VAC3 | barcode06 | 2018 | 11 | Virginia | C3 | 608,888 | 199,094 | 32.7 |
| | | 1811_VAM1 | barcode07 | 2018 | 11 | Virginia | M1 | 399,416 | 111,706 | 27.97 |
| | | 1811_VAM2 | barcode08 | 2018 | 11 | Virginia | M2 | 402,382 | 129,405 | 32.16 |
| | | 1811_VAM3 | barcode09 | 2018 | 11 | Virginia | M3 | 255,650 | 167,883 | 65.67 |
| | | 1811_IL1 | barcode10 | 2018 | 11 | Illinois | IL1 | 359,734 | 103,433 | 28.75 |
| | | 1811_IL2 | barcode11 | 2018 | 11 | Illinois | IL2 | 514,291 | 116,361 | 22.63 |
| | | 1811_IL3 | barcode12 | 2018 | 11 | Illinois | IL3 | 182,892 | 43,556 | 23.82 |
| | 2 | 1811_SC1 | barcode01 | 2018 | 11 | South Carolina | SC1 | 326,741 | 72,278 | 22.12 |
| | | 1811_SC2 | barcode02 | 2018 | 11 | South Carolina | SC2 | 699,492 | 99,627 | 14.24 |
| | | 1811_SC3 | barcode03 | 2018 | 11 | South Carolina | SC3 | 370,386 | 108,194 | 29.21 |
| | | 1811_NYNE | barcode04 | 2018 | 11 | New York | NYNE | 422,761 | 60,853 | 14.39 |
| | | 1811_NYNW | barcode05 | 2018 | 11 | New York | NYNW | 368,033 | 60,273 | 16.38 |
| | | 1811_NYSE | barcode06 | 2018 | 11 | New York | NYSE | 305,199 | 30,750 | 10.08 |
| | | 1601_VAC1 | barcode07 | 2016 | 1 | Virginia | C1 | 342,154 | 84,099 | 24.58 |
| | | 1601_VAC2 | barcode08 | 2016 | 1 | Virginia | C2 | 368,717 | 48,635 | 13.19 |
| | | 1601_VAC3 | barcode09 | 2016 | 1 | Virginia | C3 | 432,612 | 90,362 | 20.89 |
| | | 1601_VAM1 | barcode10 | 2016 | 1 | Virginia | M1 | 364,527 | 21,609 | 5.93 |
| | | 1601_VAM2 | barcode11 | 2016 | 1 | Virginia | M2 | 288,557 | 80,257 | 27.81 |
| | | 1601_VAM3 | barcode12 | 2016 | 1 | Virginia | M3 | 370,746 | 55,479 | 14.96 |

Table S2. Mean differences of alpha diversity measurements between two seasons by year and state. Tukey HSD was used to test the significance of each pairwise comparison

| Year | Measurement | State | Early summer (ES) | Late fall (LF) | ES-LF | *p*-value |
|---|---|---|---|---|---|---|
| 2017 | Observed OTU richness | California | 2622 | 2157 | 465 | 0.0614 |
| | | Illinois | 2750 | 2513 | 191 | 0.9236 |
| | | New York | 2131 | 2367 | -235 | 0.6724 |
| | | South Carolina | 2601 | 2218 | 384 | 0.1991 |
| | | Virginia | 2047 | 1780 | 267 | 0.2664 |
| | Shannon index | California | 6.51 | 6.13 | 0.372 | 0.0360 |
| | | Illinois | 6.64 | 6.45 | 0.193 | 0.6953 |
| | | New York | 5.93 | 6.28 | -0.347 | 0.0247 |
| | | South Carolina | 6.43 | 6.14 | 0.288 | 0.1949 |
| | | Virginia | 5.96 | 5.87 | 0.098 | 0.9509 |
| 2018 | Observed OTU richness | California | 2764 | 2455 | 310 | 0.9772 |
| | | Illinois | 2687 | 2560 | 127 | 1.0000 |
| | | New York | 1912 | 3104 | 1193 | 0.0014 |
| | | South Carolina | 2316 | 2536 | 220 | 0.9980 |
| | | Virginia | 1338 | 1876 | 538 | 0.1946 |
| | Shannon index | California | 6.58 | 6.42 | 0.162 | 0.9601 |
| | | Illinois | 6.69 | 6.53 | 0.157 | 0.9667 |
| | | New York | 6.36 | 6.79 | -0.431 | 0.0279 |
| | | South Carolina | 6.36 | 6.26 | 0.097 | 0.9989 |
| | | Virginia | 5.86 | 5.92 | -0.058 | 0.9997 |

Significant *p*-values are shaded gray

Table S3. The five most abundant bacterial orders in garden soils by state, year, and season

| | 2017 | | | | 2018 | | | |
|---|---|---|---|---|---|---|---|---|
| **State** | **Early summer** | **%** | **Late fall** | **%** | **Early summer** | **%** | **Late fall** | **%** |
| California | *Burkholderiales* | 17.7 | *Rhizobiales* | 18.0 | *Burkholderiales* | 20.7 | *Rhizobiales* | 17.1 |
| | *Rhizobiales* | 14.5 | *Burkholderiales* | 16.6 | *Rhizobiales* | 14.1 | *Burkholderiales* | 16.0 |
| | *Chitinophagales* | 5.7 | *Vicinamibacterales* | 6.2 | *Xanthomonadales* | 5.9 | *Vicinamibacterales* | 5.7 |
| | *Vicinamibacterales* | 5.5 | *Acidobacteriales* | 4.7 | *Vicinamibacterales* | 5.3 | *Chitinophagales* | 4.0 |
| | *Xanthomonadales* | 4.5 | *Chitinophagales* | 3.7 | *Chitinophagales* | 4.8 | *Xanthomonadales* | 3.3 |
| Illinois | *Burkholderiales* | 19.8 | *Burkholderiales* | 17.8 | *Burkholderiales* | 20.0 | *Burkholderiales* | 21.3 |
| | *Rhizobiales* | 13.5 | *Rhizobiales* | 15.7 | *Rhizobiales* | 11.0 | *Rhizobiales* | 14.3 |
| | *Chitinophagales* | 6.4 | *Vicinamibacterales* | 7.7 | *Vicinamibacterales* | 5.3 | *Vicinamibacterales* | 7.7 |
| | *Vicinamibacterales* | 4.5 | *Chitinophagales* | 5.5 | *Chitinophagales* | 4.9 | *Chitinophagales* | 3.4 |
| | *Xanthomonadales* | 3.3 | *Gammaproteobacteria* | 1.8 | *Pseudomonadales* | 4.6 | *Pseudomonadales* | 2.6 |
| New York | *Rhizobiales* | 15.7 | *Burkholderiales* | 17.8 | *Rhizobiales* | 18.6 | *Rhizobiales* | 17.6 |
| | *Xanthomonadales* | 12.9 | *Rhizobiales* | 16.5 | *Burkholderiales* | 13.9 | *Burkholderiales* | 11.1 |
| | *Acidobacteriales* | 12.5 | *Vicinamibacterales* | 6.9 | *Vicinamibacterales* | 5.0 | *Sphingomonadales* | 5.6 |
| | *Burkholderiales* | 8.9 | *Chitinophagales* | 2.9 | *Sphingomonadales* | 4.6 | *Enterobacterales* | 4.9 |
| | *Chitinophagales* | 4.7 | *Gemmatimonadales* | 2.1 | *Xanthomonadales* | 3.4 | *Xanthomonadales* | 3.0 |
| South Carolina | *Rhizobiales* | 15.1 | *Burkholderiales* | 21.1 | *Rhizobiales* | 21.0 | *Rhizobiales* | 20.1 |
| | *Burkholderiales* | 14.0 | *Rhizobiales* | 16.5 | *Burkholderiales* | 14.0 | *Burkholderiales* | 15.4 |
| | *Vicinamibacterales* | 7.7 | *Vicinamibacterales* | 8.6 | *Vicinamibacterales* | 7.2 | *Vicinamibacterales* | 11.3 |
| | *Chitinophagales* | 5.3 | *Sphingomonadales* | 2.6 | *Chitinophagales* | 3.0 | *Sphingomonadales* | 2.5 |
| | *Bacillales* | 5.0 | *Chitinophagales* | 2.5 | *Polyangiales* | 2.3 | *Chitinophagales* | 2.0 |
| Virginia | *Rhizobiales* | 21.0 | *Rhizobiales* | 16.8 | *Burkholderiales* | 16.4 | *Rhizobiales* | 21.6 |
| | *Burkholderiales* | 11.6 | *Burkholderiales* | 13.3 | *Rhizobiales* | 14.3 | *Burkholderiales* | 12.0 |
| | *Vicinamibacterales* | 8.4 | *Vicinamibacterales* | 9.8 | *Vicinamibacterales* | 8.7 | *Vicinamibacterales* | 8.1 |
| | *Bacillales* | 4.3 | *Chitinophagales* | 3.4 | *Chitinophagales* | 3.8 | *Gaiellales* | 4.4 |
| | *Gaiellales* | 2.9 | *Gaiellales* | 3.1 | *Rokubacteriales* | 2.7 | *Solirubrobacterales* | 2.5 |

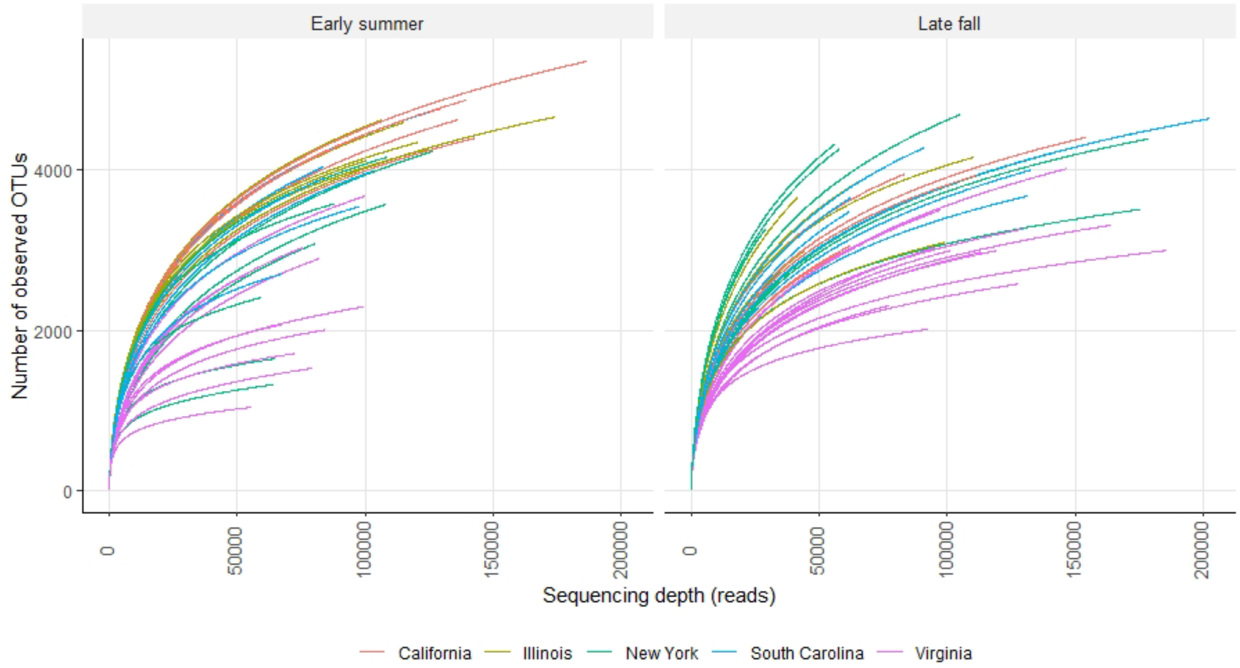


Figure S1. Rarefaction curve of sequencing depth and OTU numbers for samples collected from California, Illinois, New York, South Carolina, and Virginia in early summer and late fall on the left and right, respectively.

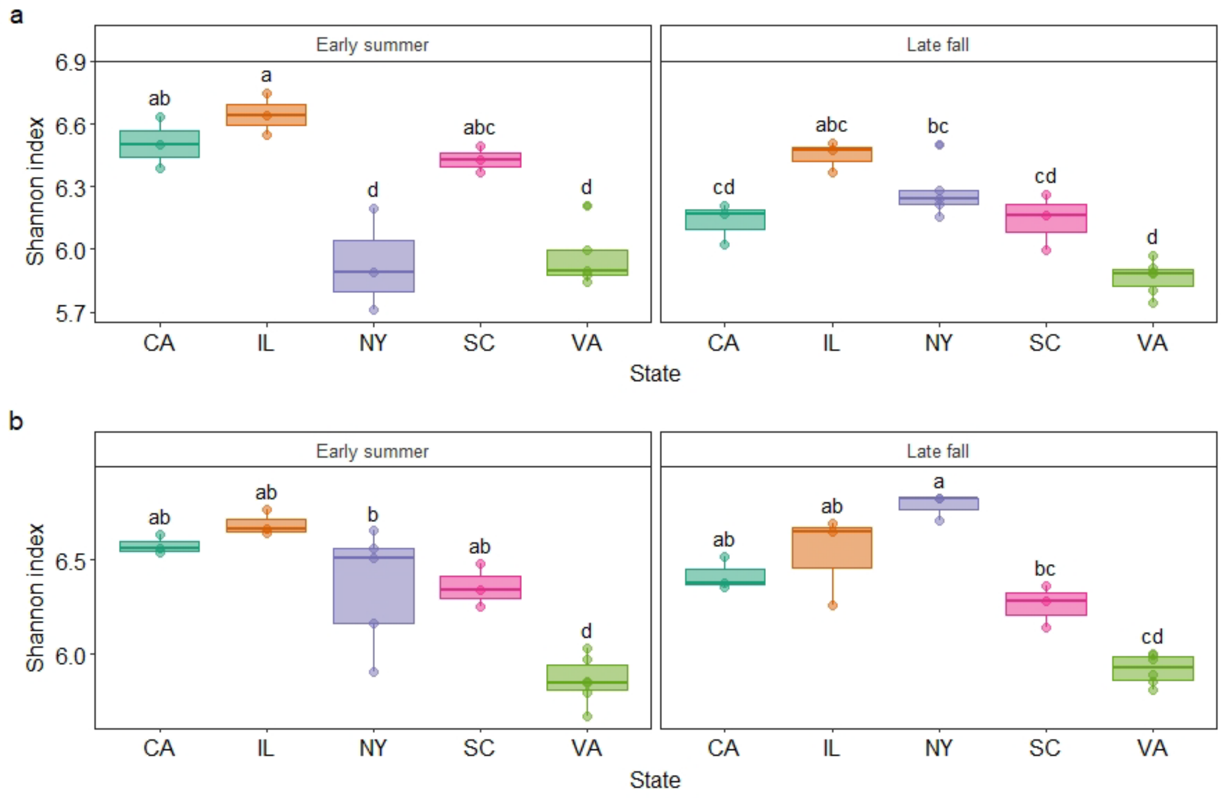


Figure S2. Shannon index of the early summer and late fall season samples collected from California (CA), Illinois (IL), New York (NY), South Carolina (SC), and Virginia (VA) in 2017 (**a**) and 2018 (**b**). Boxes topped by completely different letter(s) within each year and season differed according to Tukey HSD post hoc test at $p$=0.05.

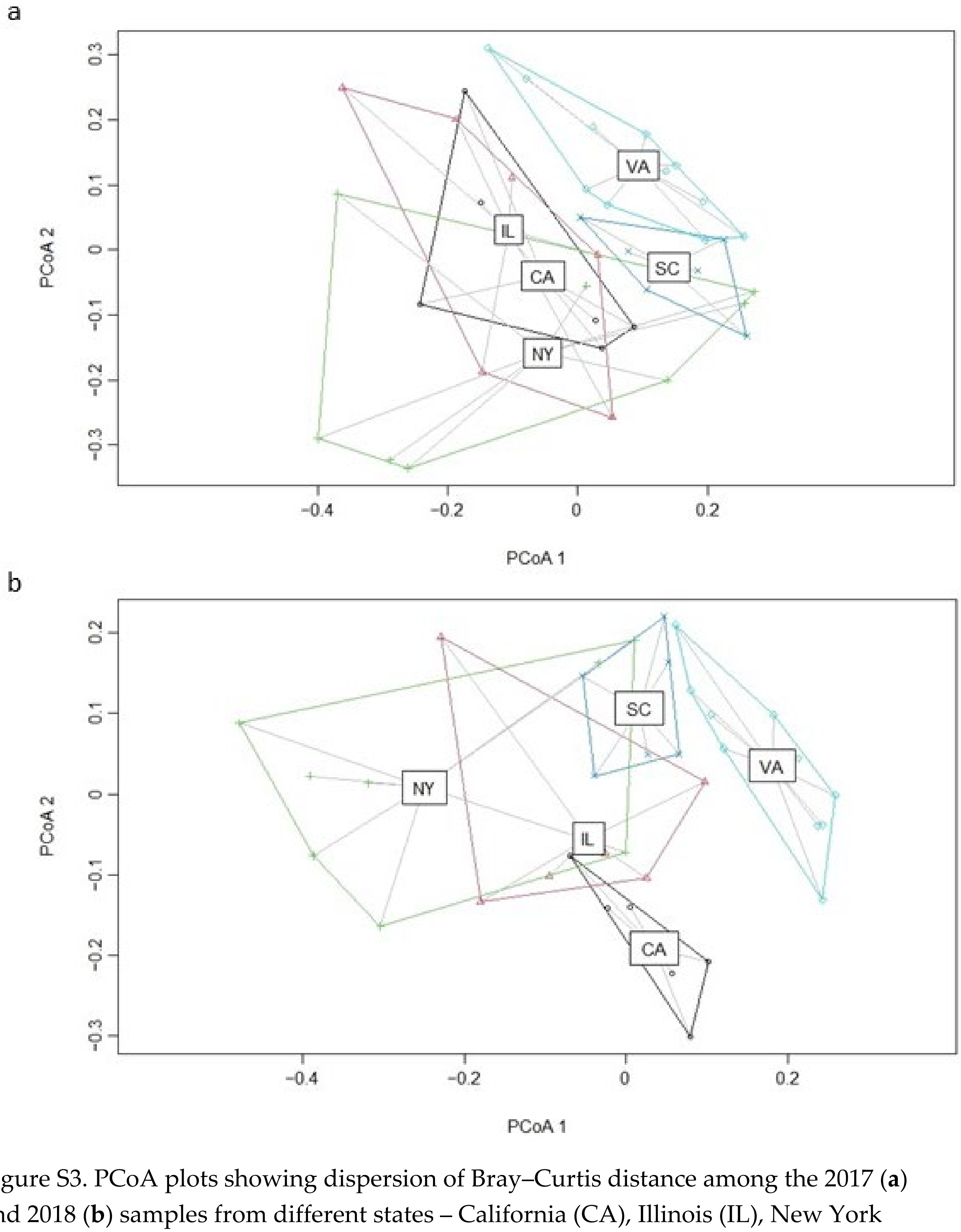


Figure S3. PCoA plots showing dispersion of Bray–Curtis distance among the 2017 (**a**) and 2018 (**b**) samples from different states – California (CA), Illinois (IL), New York (NY), South Carolina (SC) and Virginia (VA). Sample centroids are labeled.

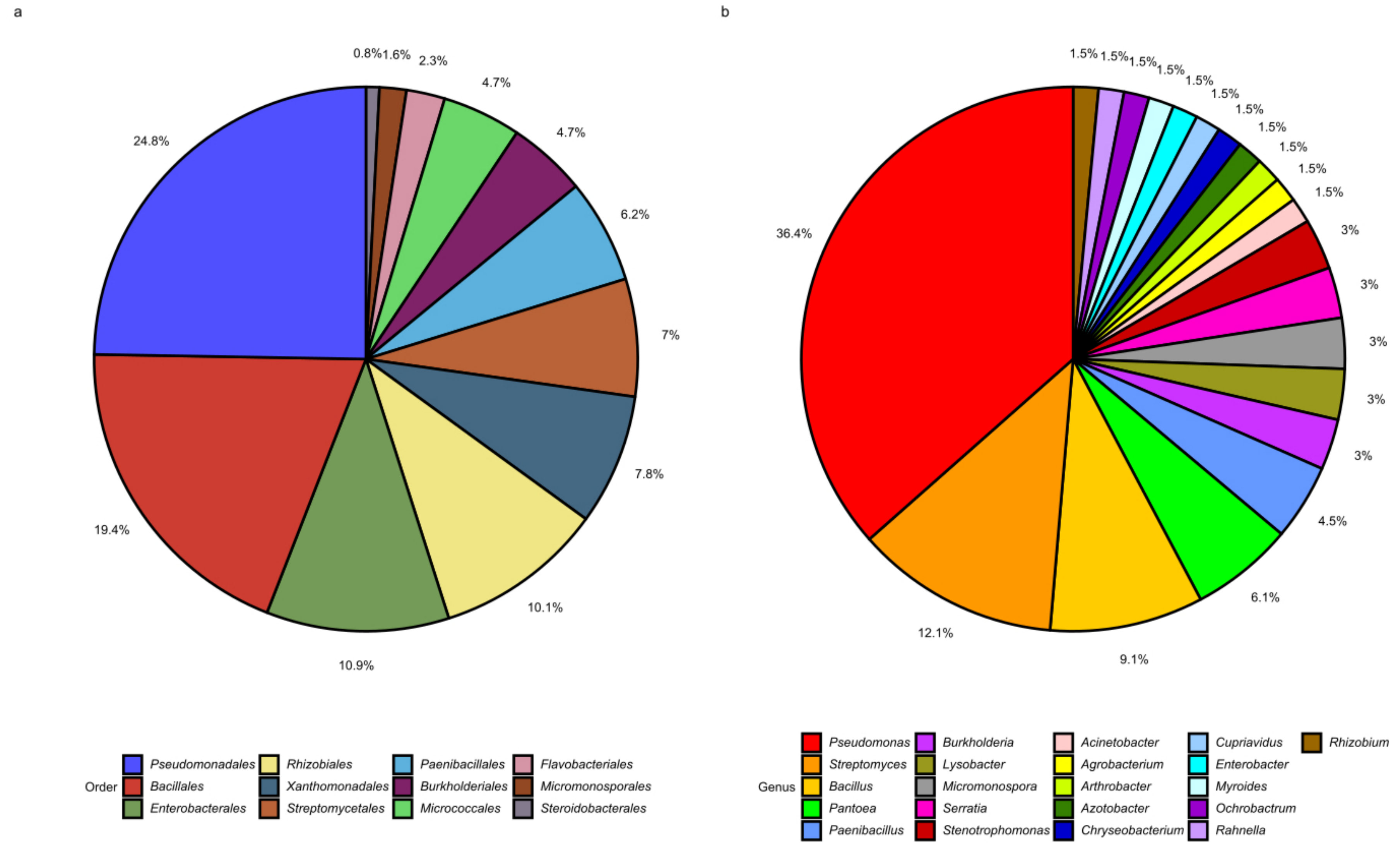


Figure S4. Pie chart showing the relative abundance of twelve bacterial orders (a) and twenty-one genera (b) to which the 66 identified species with biocontrol potentials belong, respectively.
